# Intraocular Pressure Measurement after Penetrating Keratoplasty

**DOI:** 10.3390/diagnostics12020234

**Published:** 2022-01-19

**Authors:** Otilia-Maria Dumitrescu, Sinziana Istrate, Mioara-Laura Macovei, Alina Gabriela Gheorghe

**Affiliations:** 1 Department of Ophthalmology, Central Military Emergency University Hospital, 010825 Bucharest, Romania; otiliamariadumitrescu@gmail.com; 2 Ophthalmology Department, Emergency University Hospital, 169 Splaiul Independentei, 050098 Bucharest, Romania; sanzinici@yahoo.com; 3 Department of Ophthalmology, University of Medicine and Pharmacy “Carol Davila”, 050474 Bucharest, Romania; alina.gheorghe.g@gmail.com

**Keywords:** penetrating keratoplasty, intraocular pressure, glaucoma, Goldmann applanation tonometry, Pascal dynamic contour tonometry, Tono-Pen XL, ocular response analyzer, iCare

## Abstract

Assessing the intraocular pressure is a difficult but crucial task in the follow-up of patients that have undergone penetrating keratoplasty. Early recognition of elevated intraocular pressure and/or glaucoma and establishment of the appropriate treatment is essential to ensure the best possible visual outcome for patients dealing with this feared complication. Although Goldmann applanation tonometry is still the gold standard for measuring the intraocular pressure, its limitations in postkeratoplasty eyes, due to postoperative modified corneal morphology, have led to the search for more suitable alternatives. This review is the result of a comprehensive literature search in the MEDLINE database that aims to present glaucoma in the context of perforating keratoplasty, the corneal properties with impact on ocular pressure measurement, and the results achieved with the most important tonometers that have been studied in this pathology. Goldmann applanation tonometry remains the reference for intraocular pressure assessment even in corneas after penetrating keratoplasty. However, some promising alternatives have emerged, the most important of which are the Pascal dynamic contour tonometry, the Tono-Pen XL, the ocular response analyzer, and the iCare. All have advantages and disadvantages but have proved to be appropriate alternatives, especially in cases in which Goldmann applanation tonometry cannot be used.

## 1. Introduction

Penetrating keratoplasty (PK) is one of the oldest and most performed types of human transplantation and consists of full-thickness corneal replacement [1]. The main aim of PK is to restore vision by improving the optics of the eye [2,3]. Although in recent years, the newer lamellar techniques of corneal transplantation have gained increased popularity, PK is still a widely used procedure in cases of blinding corneal diseases [4,5]. The current place of PK among the corneal transplantation techniques varies in different parts of the world. In Colombia (South America), according to a study conducted between 2012 and 2016, PK represented 73.3% of all the corneal transplants performed [6], while in Europe, according to the report by the European Cornea and Cell Transplantation Registry published in 2021, which included 10 European countries, PK (accounting for 30% of all corneal transplants) was in second place after Descemet stripping automated endothelial keratoplasty (DSAEK) [3]. This difference may reflect the availability of the newer transplant techniques, as well as differences in the indications for corneal grafting [3,6]. The same European study found that PK is the technique of choice in the case of keratoconus, infectious keratitis, and regrafts [3].

One of the most feared complications after PK is glaucoma, as it is associated with important visual morbidity and is a major cause of blindness in these patients [7,8]. This is why early recognition of high intraocular pressure (IOP) is important after PK, in order to promptly institute the appropriate treatment [9]. However, IOP measurement in post-PK eyes is difficult [10], because of the changes in corneal architecture that occur postoperatively. The longstanding role of the Goldmann applanation tonometry (GAT) as the “gold standard” is being questioned in the case of these patients. Therefore, there is an ongoing search for alternative modalities of IOP measurement that are more suited for eyes that have undergone full-thickness corneal transplantation.

## 2. Materials and Methods

We conducted an extensive literature search in the MEDLINE electronic database, using the PubMed interface. The search process comprised the following word combinations: “intraocular pressure” AND either “penetrating keratoplasty” or “corneal transplantation”. Inclusion criteria consisted of articles written in English, regarding human pathology and subjects, that had appeared before November 2021. The title and abstract were evaluated, and we subsequently retained those studies that described and/or compared methods of IOP measurement used after penetrating keratoplasty, as well as studies that explored the related subjects of elevated IOP and glaucoma in the context of corneal transplantation and that of corneal properties before and after penetrating keratoplasty. We also obtained some additional references from the reference lists of the already retained studies. We excluded studies that were duplicates of the previously selected ones, that were not relevant to the chosen topic, as well as editorials, letters to editors, comments and conference presentations, and studies on animal models. After applying the inclusion and exclusion criteria to the initial search result, we retained 55 articles, dating from 1987 to 2021.

## 3. Literature Review

### 3.1. Ocular Hypertension and Glaucoma after Penetrating Keratoplasty

Glaucoma is the most important cause of irreversible vision loss after PK [7]. Vision loss is the result of progressive and permanent optic nerve damage [11] and of graft failure secondary to endothelial cell loss [8,9,12,13]. Moreover, treatment of glaucoma after PK is usually more challenging, with many patients requiring surgery, which, in turn, increases the risk of graft failure [9,14].

After PK, glaucoma has an incidence between 8.7% and 53% [13,15,16,17]. Some of the most important mechanisms responsible for IOP elevation after PK are tight sutures at the graft–host interface, changes at the level of the iridocorneal angle (collapse of the trabecular meshwork, angle distortion), postoperative intraocular inflammation, with or without peripheral anterior synechiae formation, or posterior synechiae with subsequent pupillary block, the persistence of viscoelastic substance or vitreous in the angle and long-term steroid therapy [5,10,18,19,20,21].

The most important risk factor associated with ocular hypertension after PK is a preoperative diagnosis of glaucoma [5,11,13,16,22]. Other major risk factors are aphakia, pseudophakia, anterior chamber IOL, the association of PK with another surgery, the presence of peripheral anterior synechiae, preoperative inflammatory states (such as a recent history of trauma, corneal abscess, graft rejection, or bullous keratopathy), preoperative scarring, the presence of an adherent leukoma, and postoperative treatment with prednisolone acetate 1% [5,11,13,16,22,23,24]. There is a lower risk for developing postoperative glaucoma if the indication for PK is keratoconus or corneal dystrophies, as opposed to other indications [16].

Most cases of glaucoma after PK are diagnosed in the first year following surgery [13]. Therefore, a close and careful follow-up is warranted, in order to make an early diagnosis and institute appropriate treatment. Goldmann applanation tonometry (GAT) is regarded as the gold standard for IOP measurement [25]. However, corneal morphology appears to influence GAT, which results in measurement variations even in normal corneas [26]. Post-PK, corneal architecture is sensibly different from that of a normal, intact cornea, which may render GAT unreliable in these eyes [27]. Other pathologies associated with an abnormal corneal architecture also make IOP estimation difficult. Such is the case in conjunctival burns, ocular pemphigoid [28] and after surgery involving the cornea (conjunctival flap [29], corneal crosslinking [30], and refractive surgery [31,32]). This is why efforts have been made to find more suitable alternatives to GAT in eyes with abnormal corneas, and in particular in postkeratoplasty ones.

### 3.2. Corneal Properties with Impact on IOP Measurement and Their Evolution after PK

#### 3.2.1. Central Corneal Thickness (CCT)

Corneal thickness has long been considered to influence IOP measurement [26,33]. A meta-analysis by Doughty et al. that included approximately 600 CCT datasets found that the CCT in normal white adults is expected to be within 11.6% of 535 µm [33]. Post-PK, reported CCT values varied in different studies, as can be seen in Table 1. CCT appears to have higher values in post-PK eyes, compared with normal corneas, but no statistical significance has been proven [34].

Doughty et al. [33] calculated that a difference of 10% in CCT may result in a 3.4 ± 0.9 mm Hg difference in measured IOP by applanation tonometry [33]. However, the authors also concluded that this difference has no clinical significance for IOP measurement over a wide range of CCT in normal eyes. Most studies did not find a statistically significant influence of CCT on IOP measurement in eyes that have undergone PK, regardless of the instrument used [2,19,34,35,38,42,43,44]. Few studies reported otherwise [39,41,45], and the correlation between CCT and IOP measurement was weak [45]. Nevertheless, when IOP measurements are elevated or borderline, especially in abnormal corneas, it is advisable to measure and take into account the CCT [33].

#### 3.2.2. Corneal Astigmatism

Post-PK corneas usually display important astigmatism, with mean values ranging from 4.73 to 5.88 D [19,35,38,40]. Studies have shown that IOP measurement is independent of corneal astigmatism [35,38,43], especially in the early postoperative period (less than one year) [35]. High corneal astigmatism requires an adaptation of the IOP measuring technique when using GAT, which consists of making two separate measurements—one with the prisms oriented vertically, and the other with the prisms oriented horizontally, the resultant final IOP being the mean between these two measurements [26,35].

#### 3.2.3. Corneal Curvature (CC)

The mean corneal curvature following PK has shown similar values throughout different studies: 45.9 ± 2.3 diopters (D) [26]; 45.5 ± 2.2 D [26]; 45.4 ± 2.7 D [38]. There is conflicting evidence regarding the influence of corneal curvature on IOP measurement post-PK. Ceruti et al. [43] found that this influence was not significant. However, in other studies, positive correlations (i.e., a steeper cornea results in a hyperestimation of IOP [41]) have been reported between CC and diverse IOP measuring techniques: the Pascal dynamic contour tonometer (PDCT) [41], Goldmann applanation tonometry (GAT) [41], the iCare [38] and non-contact tonometry [45].

#### 3.2.4. Corneal Hysteresis (CH) and Corneal Resistance Factor (CRF)

Corneal rigidity is another element suspected to influence IOP measurement and is the result of the interplay of numerous factors, such as corneal thickness and hydration. Corneal hysteresis and the corneal resistance factor are two parameters that reflect the viscoelastic properties and, thus, the rigidity of the cornea. These parameters are calculated by using the ocular response analyzer (ORA; manufactured by Reichert Ophthalmic Instruments, Buffalo, NY, USA), an instrument designed to explore the biomechanical properties of the cornea. Corneal pathology often results in altered corneal properties. For example, ectatic disorders and Fuchs endothelial dystrophy are associated with a significantly lower CH when compared with normal corneas [46]. The study of Yenerel et al. found that PK for the treatment of ectatic disorders manages to improve, although not return to normal, the biomechanical properties of the cornea [27]. CH and CRF were found to be significantly higher in normal versus post-PK eyes [34,47].

Factors that contribute to the postoperative biomechanical properties are the remaining peripheral rim of recipient cornea, wound healing at the graft–host interface, and the corneal button itself [2,27,48]. It appeared beneficial to use a slightly oversized graft (>0.5 mm), for several reasons [2]. Firstly, corneal biomechanics are improved, with higher postoperative CH and CRF. Secondly, a more peripherally placed graft–host interface may provide better refractive results due to lower grade astigmatism. Moreover, a larger graft brings a larger contingent of endothelial cells, thus helping graft survival [2].

CH and CRF are not influenced by corneal astigmatism [47] or by CCT in post-PK eyes [34]. Their impact on IOP measurement has also been explored. CH and CRF have both been shown to correlate with the IOP measurements obtained using the ORA [19,42,47], while CH also appeared to correlate with GAT [42].

#### 3.2.5. The Presence of Corneal Sutures

Many studies excluded post-PK eyes in which sutures were still present, as, presumably, sutures at the graft–host interface may influence corneal properties such as astigmatism and corneal curvature and may act as confounding factors. However, the studies that explored the influence the sutures have on IOP measurement found that their presence did not significantly impact IOP measurements [19,35,43].

### 3.3. Methods of IOP Measurement

#### 3.3.1. Goldmann Applanation Tonometry

The Goldmann applanation tonometer (Haag-Streit AG, Koeniz, Switzerland) is a slit-lamp-mounted instrument that uses the Imbert–Fick principle to determine the IOP value [49]. Due to its calibration, GAT provides an accurate measurement of the IOP value for a CCT of around 520 μm [26,35]. GAT characteristically underestimates IOP in thinner corneas, while overestimating it in thicker ones [26]. This has been demonstrated in patients that had undergone photorefractive surgery and for whom, due to the thinner cornea, GAT provided lower measurements postoperatively [32]. Algorithms have been developed that calculate an IOP adjusted for the CCT value. Apart from corneal thickness, important astigmatism, an irregular corneal surface, abnormal corneal rigidity, and the altered properties at the graft–host interface, all of which may be found in post-PK corneas, also seem to impact IOP measurement by GAT, reducing its accuracy [2,34]. Underestimation in edematous corneas and overestimation in cases of corneal scarring has also been noted with GAT [50]. In post-PK eyes, repeated GAT measurements may be detrimental for the fragile transplanted epithelium [51]. However, to this day, GAT remains the “gold standard” for IOP measurements to which all the other methods are being compared.

#### 3.3.2. Pascal Dynamic Contour Tonometry (PDCT)

Pascal dynamic contour tonometry (Swiss Microtechnology AG, Port, Switzerland) is a more recently introduced contact, non-applanating method, also mounted on the slit lamp [26,41]. PDCT measurements appear to be highly consistent with GAT measurements [10,41,43]. However, there is a significant overestimation of the IOP value using PDCT when compared with GAT; the mean difference between the two methods is shown in Table 2 [10,35,40,41,43]. This difference seems to be less important for lower IOPs [43], and it has been suggested that PDCT overestimates IOP at higher values and underestimates it at low values [35]. PDCT measurements were not significantly influenced by CCT [26,35,52] and showed much less variation with CCT when compared with GAT [52,53]. PDCT also appeared to be independent of corneal astigmatism [35]. PDCT was superior to GAT with regard to the rate of successful measurements (difference not statistically significant) [10] and showed better consistency in measurements when comparing patients preoperatively and postoperatively [37,41]. It was suggested that PDCT is a suitable method for IOP measurement following PK in cases in which GAT is unreliable [35] and that it may be more reliable than GAT for measuring IOP in corneas suffering from an ectatic disease (keratoconus or cornea pellucida), even after PK has been performed, as GAT appears to underestimate the IOP in these patients [26].

#### 3.3.3. Ocular Response Analyzer

Apart from the previously discussed viscoelastic properties of the cornea, expressed as the corneal hysteresis and the corneal resistance factor, the ORA also measures two additional parameters: the Goldmann correlated intraocular pressure (IOPg), and the corneal compensated intraocular pressure (IOPcc). Both the IOPcc and IOPg correlate well with GAT, but IOPcc has significantly higher values than GAT [2,19,35]. It is unclear whether a significant difference exists between the IOPg and the GAT measurements [27,48]. Similar to PDCT, ORA has a tendency to overestimate IOP at high values and to underestimate it at low values [35].

Apart from CH and CRF, which seem to impact the values of both IOPcc and IOPg [19,42,47], IOPcc appears to be independent of most corneal properties and, thus, may provide a more accurate measurement of the IOP [2,27]. Jafarinasab et al. [48] favored the use of IOPcc over IOPg and suggested that, perhaps, a new linear calibration coefficient should be adapted for a transplanted cornea and used instead of the current coefficient (developed for normal eyes). A limitation of the ORA, especially in eyes with poor vision, as is often the case after PK, is that it requires the patient to fixate, in order to obtain a correct measurement [19].

#### 3.3.4. Tono-Pen XL

The Tono-Pen XL (Reichert, Inc., Depew, New York, NY, USA) is an applanation tonometer that uses the MacKay–Marg principle [19,25]. As the applanation area is small, this kind of tonometry appears to be more suitable for irregular corneas and for corneas with very low or very high thicknesses [54,55]. Tono-Pen XL displayed similar results to GAT in eyes after PK [35,37], with a correlation coefficient of 0.84 [39]. Shemesh et al. [39] found that 67% of Tono-Pen measurements were within ±4 mm Hg of GAT values, while Rao et al. [44] reported a closer match, with 84% of measurements being within a ±3 mm Hg interval. Other studies reported significantly higher values for Tono-Pen when compared with GAT, especially for lower IOPs [19,25,45], while Geyer et al. [25] stated that Tono-Pen overestimates IOP in a non-consistent fashion. Tono-Pen was also found to yield significantly different results, compared with PDCT, not showing consistency in preoperative versus postoperative eyes [37]. Tono-Pen XL positively correlated with CRF and inversely correlated with the time span between PK and the moment of the measurement [19]. Despite its drawbacks, Tono-Pen XL may be considered an appropriate alternative to GAT when GAT cannot be used [19,35].

#### 3.3.5. iCare

The iCare (Tiolat Oy, Helsinki, Finland) is a handheld tonometer based on the principle of rebound tonometry [38,56]. A study by Salvetat et al. showed that, although iCare and GAT measurements appeared to be highly correlated, iCare significantly underestimated the IOP in post-PK cases (−5.5 ± 3.6 mm Hg), except in the case of edematous grafts, in which the iCare overestimated the IOP value (+6.5 ± 1.9 mm Hg) [38]. Overall, for eyes after PK, there was poor agreement between the two methods and, in 57% of cases, the difference between measurements was more than 5 mm Hg. In another study that compared four methods of IOP measurement in edematous post-PK corneas and in normal eyes, iCare showed acceptable agreement with GAT in both normal and post-PK eyes. iCare showed a weak correlation with CCT and CC, and the authors concluded that it may be a good tool for IOP measurement in eyes with corneal edema [45].

#### 3.3.6. Other IOP Measuring Methods

Other commonly used tonometers (the Perkins tonometer, the Schiotz tonometer, non-contact tonometry) have not been as well investigated in post-PK eyes as the ones previously discussed. In a study by Yeh et al., non-contact tonometry (NCT) was shown to significantly overestimate the IOP and to have a poor agreement with GAT in edematous post-PK corneas [45]. NCT displayed a weak correlation with CCT and a moderate correlation with CC [42]. In another study, Jain et al. [57] investigated the use of the Schiotz tonometer in post-PK eyes and found a good correlation with GAT but with large limits of agreement. Therefore, they concluded that the Schiotz indentation tonometer cannot replace GAT in postkeratoplasty eyes. A promising alternative to GAT may be the ocular blood flow tonometer (OBF Laboratories UK, Ltd., Malmesbury, Wiltshire, UK) [44]. In the study by Rao et al. [44], the OBF showed good agreement with GAT in postkeratoplasty eyes, the mean difference between GAT and OBF being −0.68 mm Hg. The OBF did not appear to be significantly influenced by CCT, and the authors concluded that it could represent a useful alternative to GAT in post-PK eyes. However, further investigation is warranted.

Techniques bypassing the cornea, which would appear as good solutions in the case of irregular corneas, have also been explored. In one study, transpalpebral tonometry was compared with Tono-Pen XL and GAT and was shown to yield lower values. It is still uncertain whether this is related to the instrument’s unreliability or whether the IOP measured with this method is actually more accurate [39]. Magalhaes and Aldave [36] investigated a different technique, the scleral pneumatometry, in which measurements are performed at the level of the sclera. They found a strong correlation coefficient between this method and GAT, especially for inferotemporal and inferior scleral measurements. The correlation appeared even stronger when, additionally, the contralateral eye was used in the equation. These methods may prove useful in cases where corneal measurements cannot be obtained.

It is important to note that by far the most accurate method for assessing the IOP is the direct, manometric measurement and that this is the ideal standard with which all other tonometers should be compared. However, as this is an invasive technique with important associated risks, its use in clinical practice is not reasonable.

## 4. Conclusions

The role of the Goldmann applanation tonometry as the gold standard of IOP measurement is yet to be challenged, although changes in corneal architecture that occur after PK may make it less reliable in postkeratoplasty eyes. Other methods are emerging as possible alternatives to GAT. The Pascal dynamic contour tonometry, the Tono-Pen XL, the ocular response analyzer, and the iCare all appear to correlate well with GAT, even though all, apart from the iCare, tend to overestimate IOP when compared with GAT. The PDCT has shown promising results in multiple studies, having a good correlation with GAT, good consistency, and less dependence on corneal thickness and astigmatism when compared with GAT. The ORA aims at acquiring independence from most of the altered corneal properties of grafted eyes and future developments may further increase the reliability of the method. The Tono-Pen has the advantage of a small area of contact, thus proving useful in irregular corneas, and appears to yield better results later postoperatively. The iCare, although not as correlated with GAT, displayed a weak correlation with CCT and CC and thus can be useful for IOP measurement in eyes with corneal edema. There are also other tonometers that have shown sometimes promising, other times conflicting results. Although the most appropriate modality for IOP assessment in post-PK eyes remains Goldmann tonometry, its limitations in these cases point to the need for alternative choices with enhanced accessibility and ease of use in post-PK eyes.

## Figures and Tables

**Table 1:** Central corneal thickness after PK.

Study	CCT (Mean ± SD; µm)	Range (µm)	Comments
Fabian et al. [19]	585.92 ± 86.18	470–796	
Chou et al. [35]	585.0 ±149.0	N/A	
Papastergiou et al. [26]	549.0 ±27.7	503–608	PK performed for ectatic disorders
Papastergiou et al. [26]	536.0 ±45.3	475–622	PK performed for non-ectatic disorders
Magalhaes et al. [36]	576.3 ± 65.5	N/A	
Ozbek et al. [37]	482.3 ± 75.1	N/A	Grafts with edema and scars excluded
Salvetat et al. [38]	569.2 ± 50.4	478–698	
Shemesh et al. [39]	593.0 ± 94.0	441–804	
Ismail et al. [40]	525.0 ± 101.0	473–804	
Meyenberg et al. [41]	549.6 ± 33.5	393–679	

CCT—central corneal thickness; SD—standard deviation; N/A—not applicable.

**Table 2:** Mean difference between PDCT and GAT measurements in post-PK eyes.

Study	Mean PDCT-GAT Difference (mm Hg)
Kandarakis et al. [10]	+1.5
Chou et al. [35]	+2.12
Ismail et al. [40]	+2.67
Meyenberg et al. [41]	+3.1
Ceruti et al. [43]	+2.5

PDCT—Pascal dynamic contour tonometry, GAT—Goldmann applanation tonometry, PK—penetrating keratoplasty.
